# Supplementary material for: A comparative analysis of chromatin accessibility in cattle, pig, and mouse tissues
Source: BMC Genomics. 2020 Oct 7;21:698. doi: 10.1186/s12864-020-07078-9 (PMC7541309; doi:10.1186/s12864-020-07078-9)
Supplement: Supplementary file 1 — Additional file 1 Figure S1. Correlation of ATAC-seq signal in select technical replicates ATAC-seqlibraries. A) Pearson correlation of genome-wide signal (RPKM) in 500 bp windows. B) PCA of Cortex A technical replicate libraries alongside all biological replicates. C) PCA of pig technical replicate libraries alongside all biological replicates. D) Signal of cattle cortex technical and biological replicates at the STMN4 locus. E) Signal of pig cerebellum and hypothalamus technical and biological replicates at the STMN4 locus. Figure S2. Correlation of ATAC-seq signal in biological replicates. Scatterplots showing Pearson correlation of normalized genome-wide signal in 500 bp windows between biological replicates for cattle and pig tissues. Figure S3. ATAC-seq signal at TSS. Heatmaps depicting normalized ATAC-seq signal in the proximity of TSS, including 2 kb upstream and downstream, with TSS sorted by signal intensity. Figure S4. PCA of normalized ATAC-seq signal in consensus open chromatin identified in pig and cattle tissues tissues. Principal components 1, 2 and 3 are included to better visualize clustering of tissues. Figure S5. Mouse open chromatin localization and differential accessibility. A) Distribution of mouse consensus open chromatin relative to the Ensembl gene annotation (v96). B) Distribution of consensus peak activity, ranging from tissue-specific (accessible in only one tissue) to ubiquitous (accessible in all sampled tissues). Consensus peaks that were accessible in a single tissue were further broken down by tissue. Figure S6. Conservation of open chromatin in individual tissues. Titles above bar plots indicate the species that consensus peaks were identified in, followed by the species to which the consensus peak coordinates were projected to evaluate accessibility conservation in the corresponding tissue. Figure S7. Characterization of conserved open chromatin. Proportion of all consensus peaks, promoter consensus peaks (within 2 kb upstream an [file 12864_2020_7078_MOESM1_ESM.pdf]

A comparative analysis of chromatin accessibility in cattle, pig, and mouse tissues

Halstead et al.

Supplementary Figures and Tables

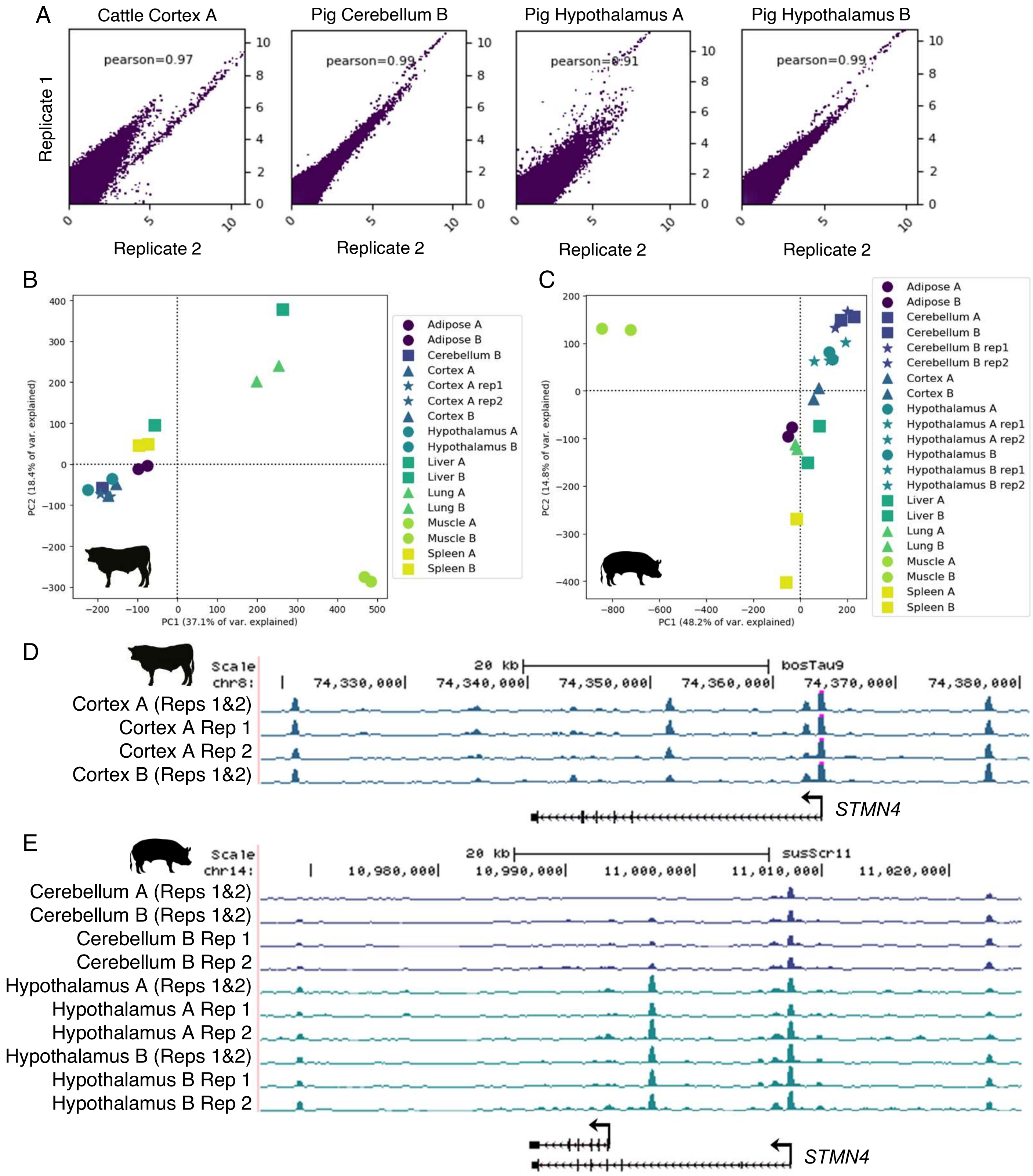

Figure S1. Correlation of ATAC-seq signal in select technical replicates ATAC-seq libraries. A) Pearson correlation of genome-wide signal (RPKM) in 500 bp windows. B) PCA of Cortex A technical replicate libraries alongside all biological replicates. C) PCA of pig technical replicate libraries alongside all biological replicates. D) Signal of cattle cortex technical and biological replicates at the *STMN4* locus. E) Signal of pig cerebellum and hypothalamus technical and biological replicates at the *STMN4* locus.

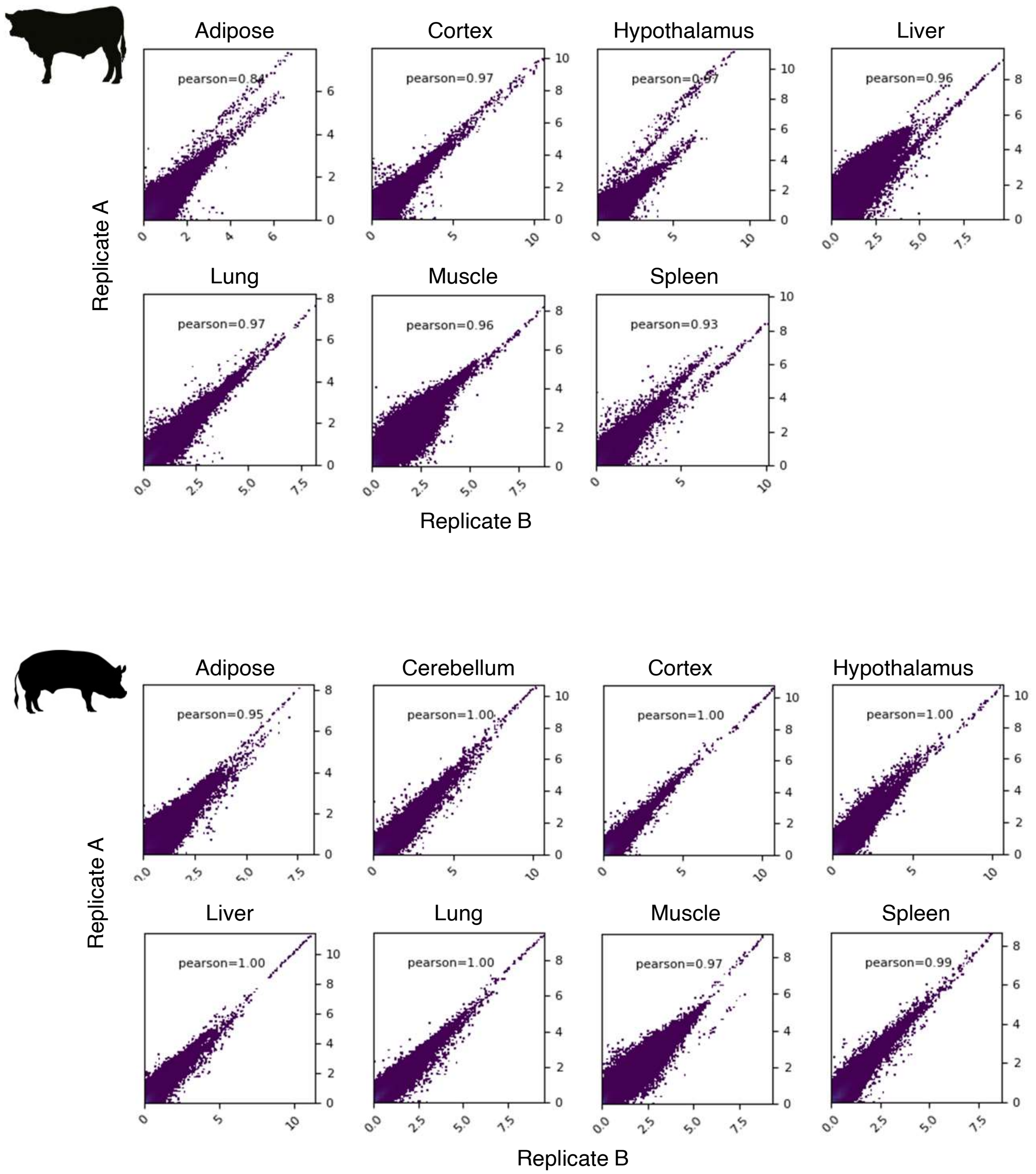

Figure S2. Correlation of ATAC-seq signal in biological replicates. Scatterplots showing Pearson correlation of normalized genome-wide signal in 500 bp windows between biological replicates for cattle and pig tissues.

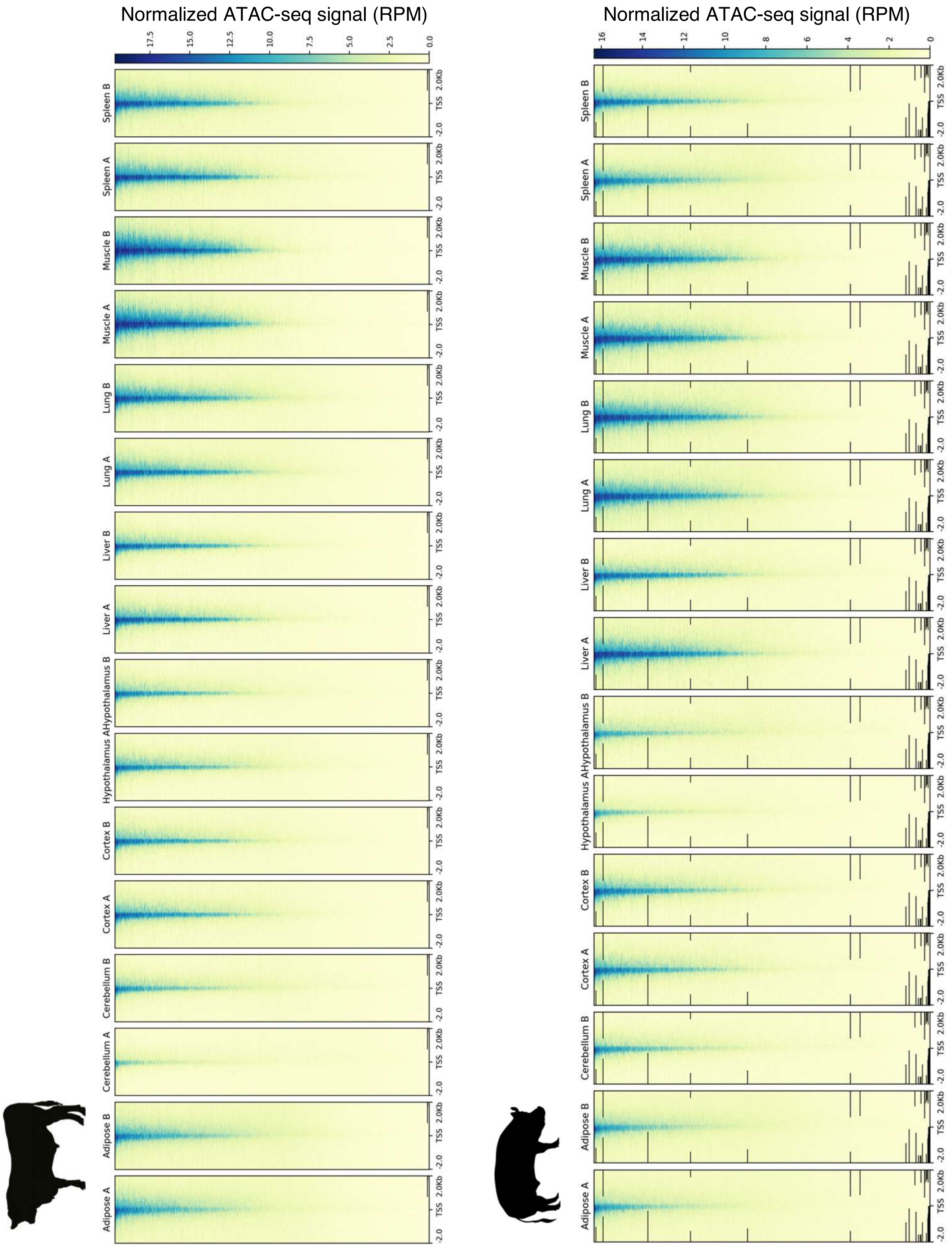

Figure S3. ATAC-seq signal at TSS. Heatmaps depicting normalized ATAC-seq signal in the proximity of TSS, including 2 kb upstream and downstream, with TSS sorted by signal intensity.

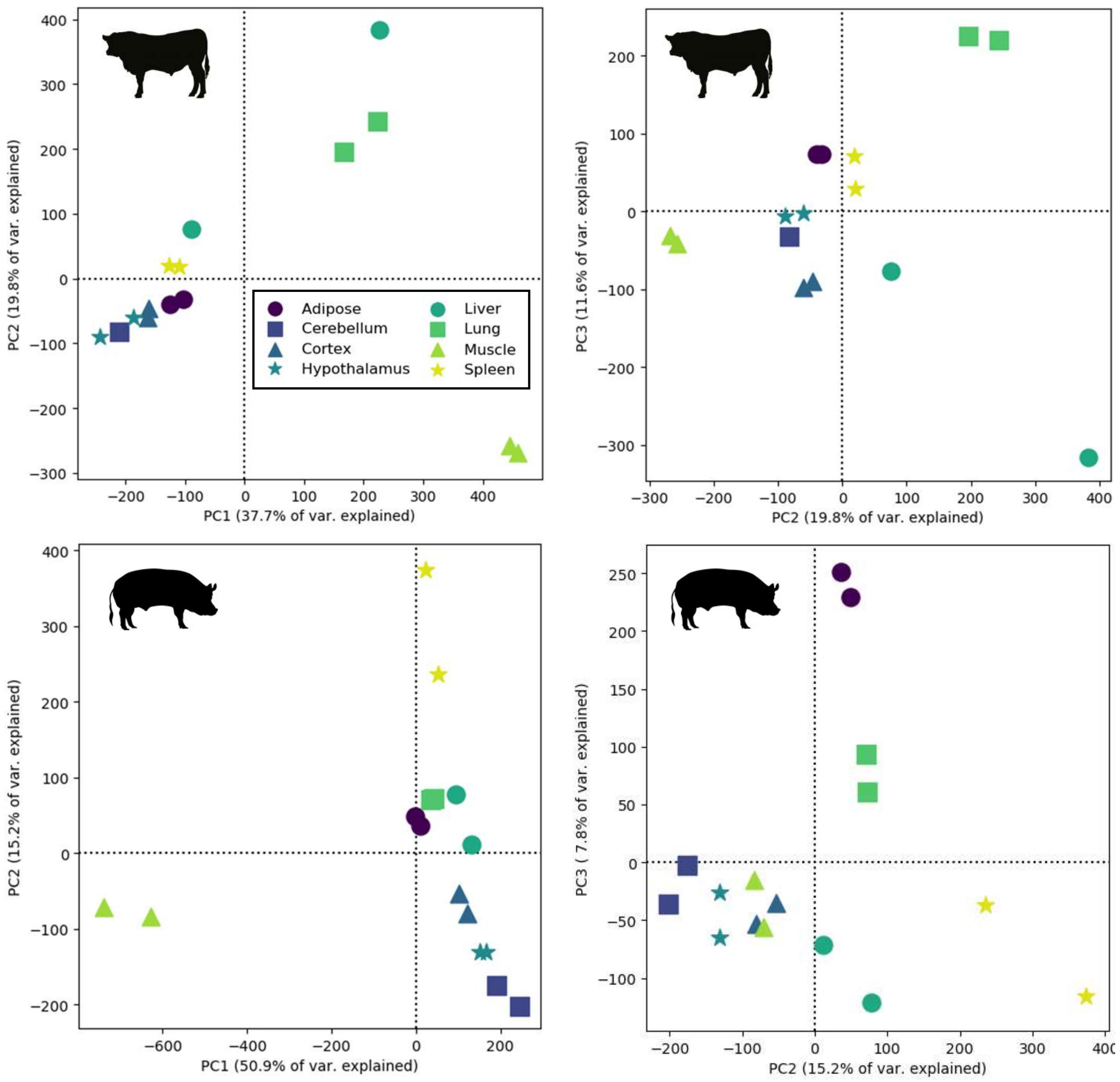

Figure S4. PCA of normalized ATAC-seq signal in consensus open chromatin identified in pig and cattle tissues tissues. Principal components 1, 2 and 3 are included to better visualize clustering of tissues.

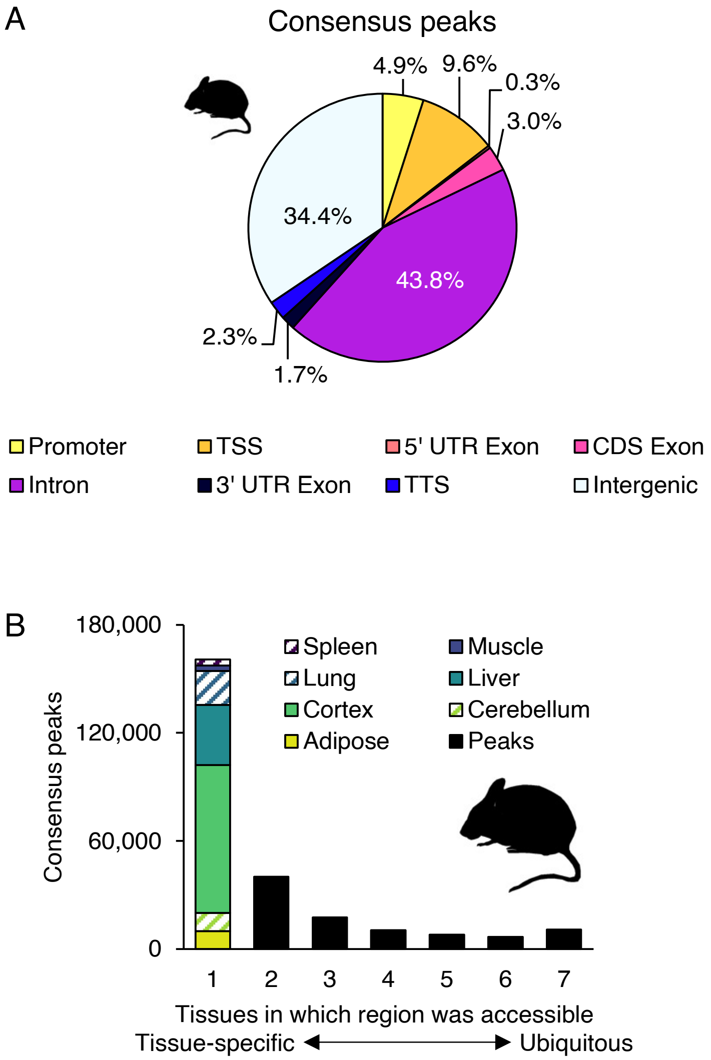

Figure S5. Mouse open chromatin localization and differential accessibility. A) Distribution of mouse consensus open chromatin relative to the Ensembl gene annotation (v96). B) Distribution of consensus peak activity, ranging from tissue-specific (accessible in only one tissue) to ubiquitous (accessible in all sampled tissues). Consensus peaks that were accessible in a single tissue were further broken down by tissue.

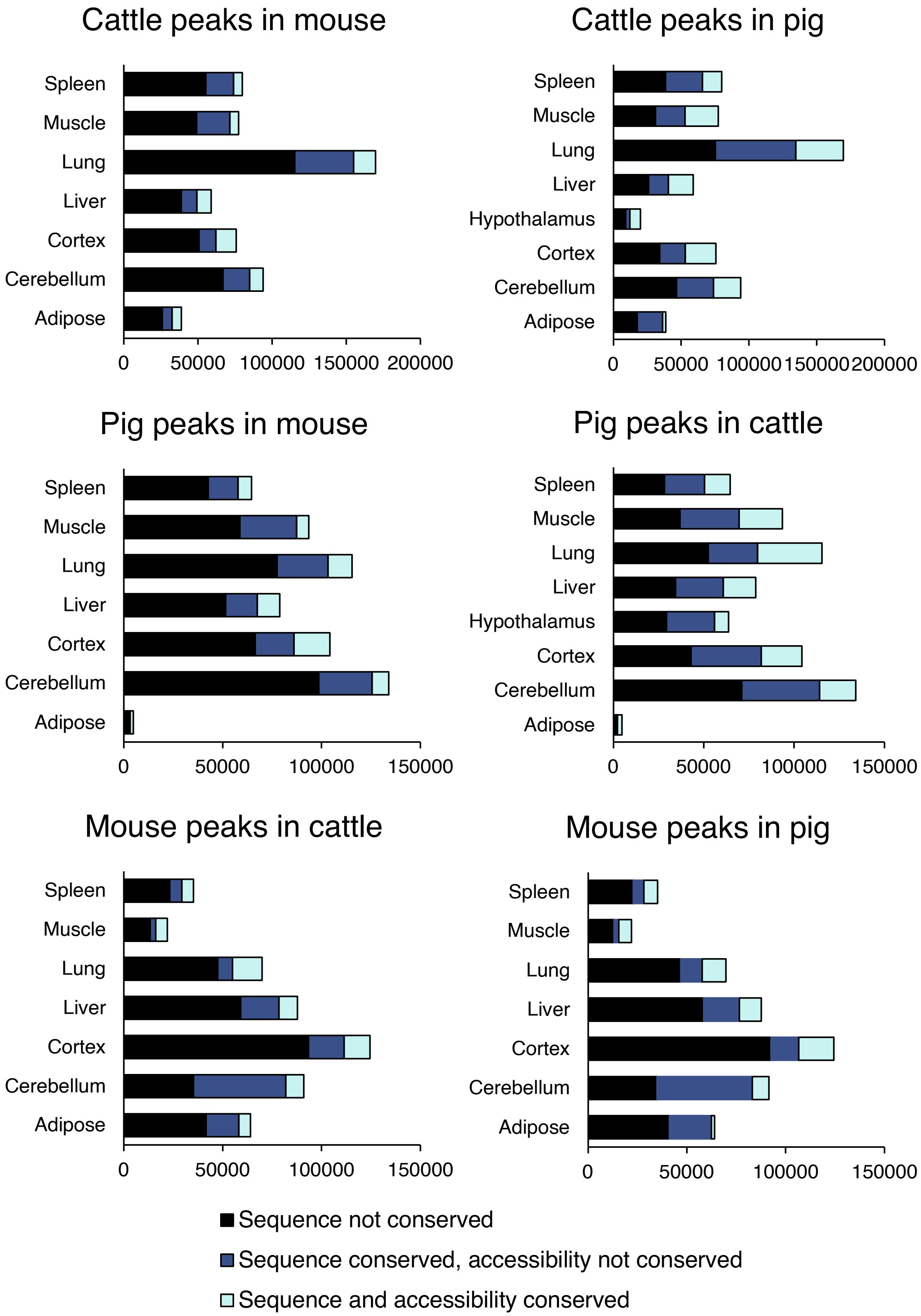

Figure S6. Conservation of open chromatin in individual tissues. Titles above bar plots indicate the species that consensus peaks were identified in, followed by the species to which the consensus peak coordinates were projected to evaluate accessibility conservation in the corresponding tissue.

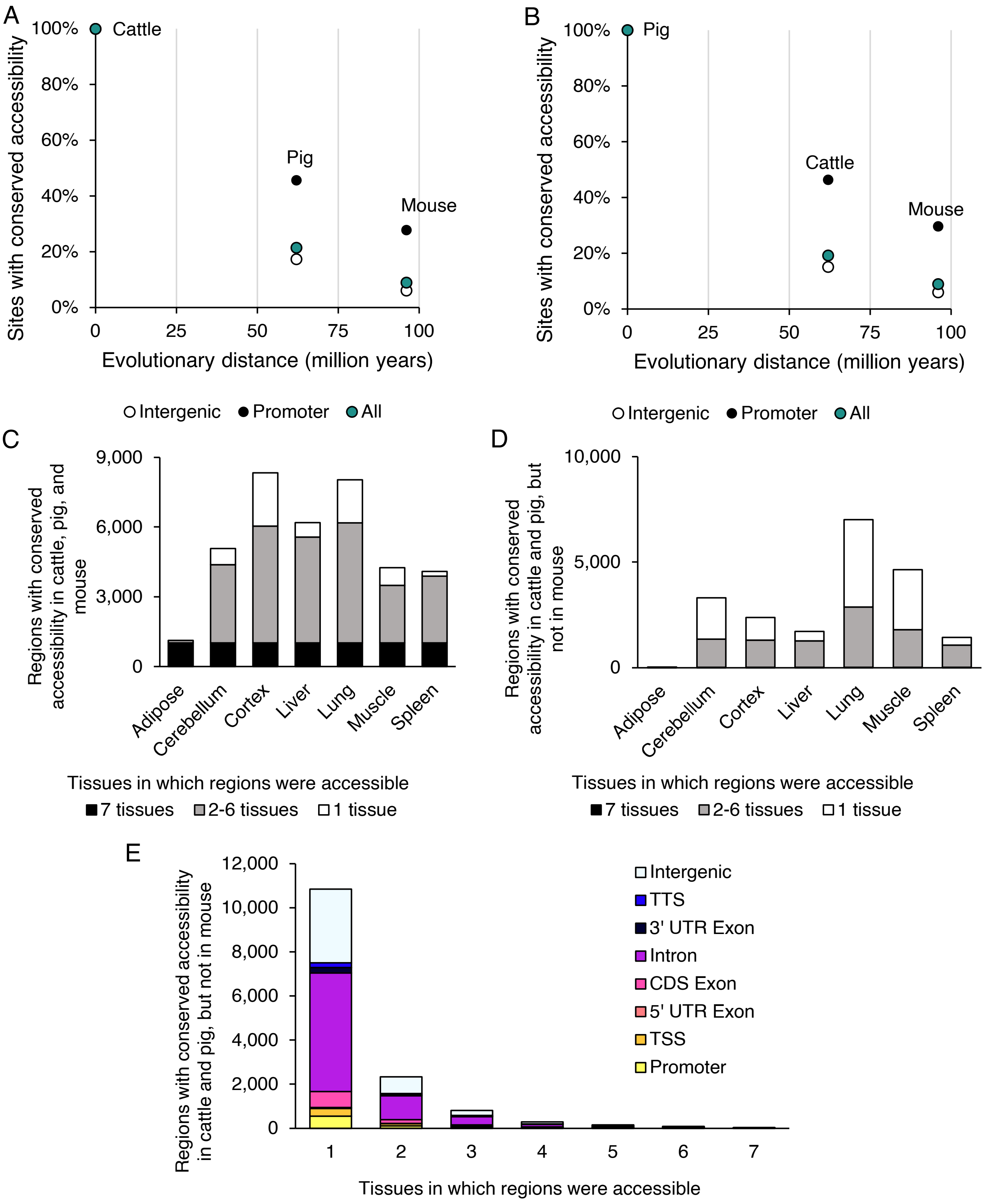

Figure S7. Characterization of conserved open chromatin. Proportion of all consensus peaks, promoter consensus peaks (within 2kb upstream and 50 bp downstream of TSS), and intergenic consensus peaks that were identified in (A) cattle or (B) pig that demonstrated both conserved sequence and accessibility in the other two species. Number of tissues in which consensus peaks demonstrated conserved accessibility in (C) all three species or (D) only in cattle and pig. E) Distribution of consensus peaks with conserved accessibility in cattle, pig, and mouse, relative to the mouse gene annotation (Ensembl v96).

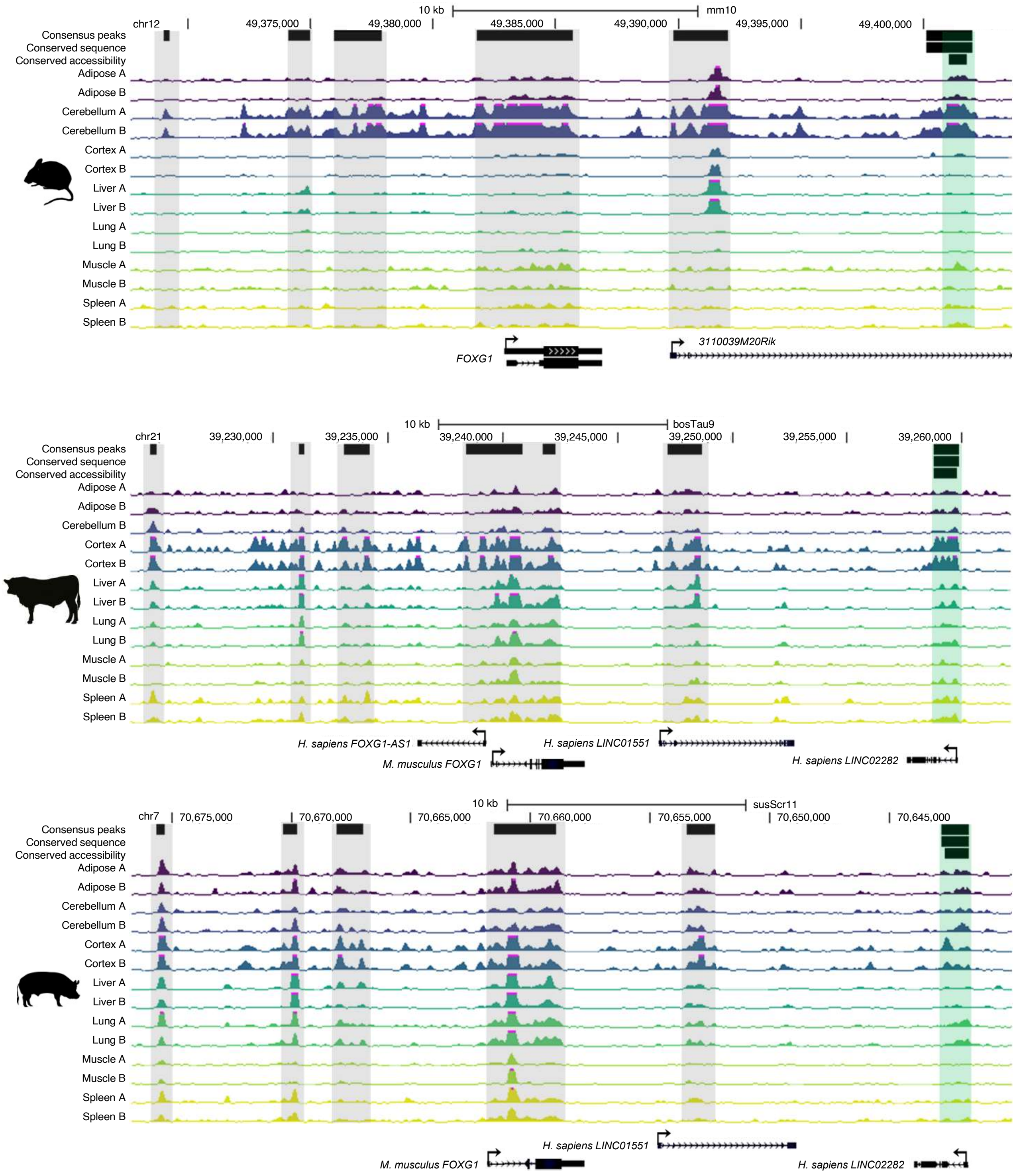

Figure S8. Positional conservation of chromatin accessibility at the *FOXG1* locus. For each species, consensus peaks, consensus peaks with conserved sequence (that could be mapped to all three species), and consensus peaks with conserved accessibility are shown. Tracks show normalized ATAC-seq signal for each sample. Conserved promoter open chromatin is highlighted in green. Consensus peaks that appear to be syntenically conserved, relative to *FOXG1*, but which could not be mapped between species, are highlighted in grey.

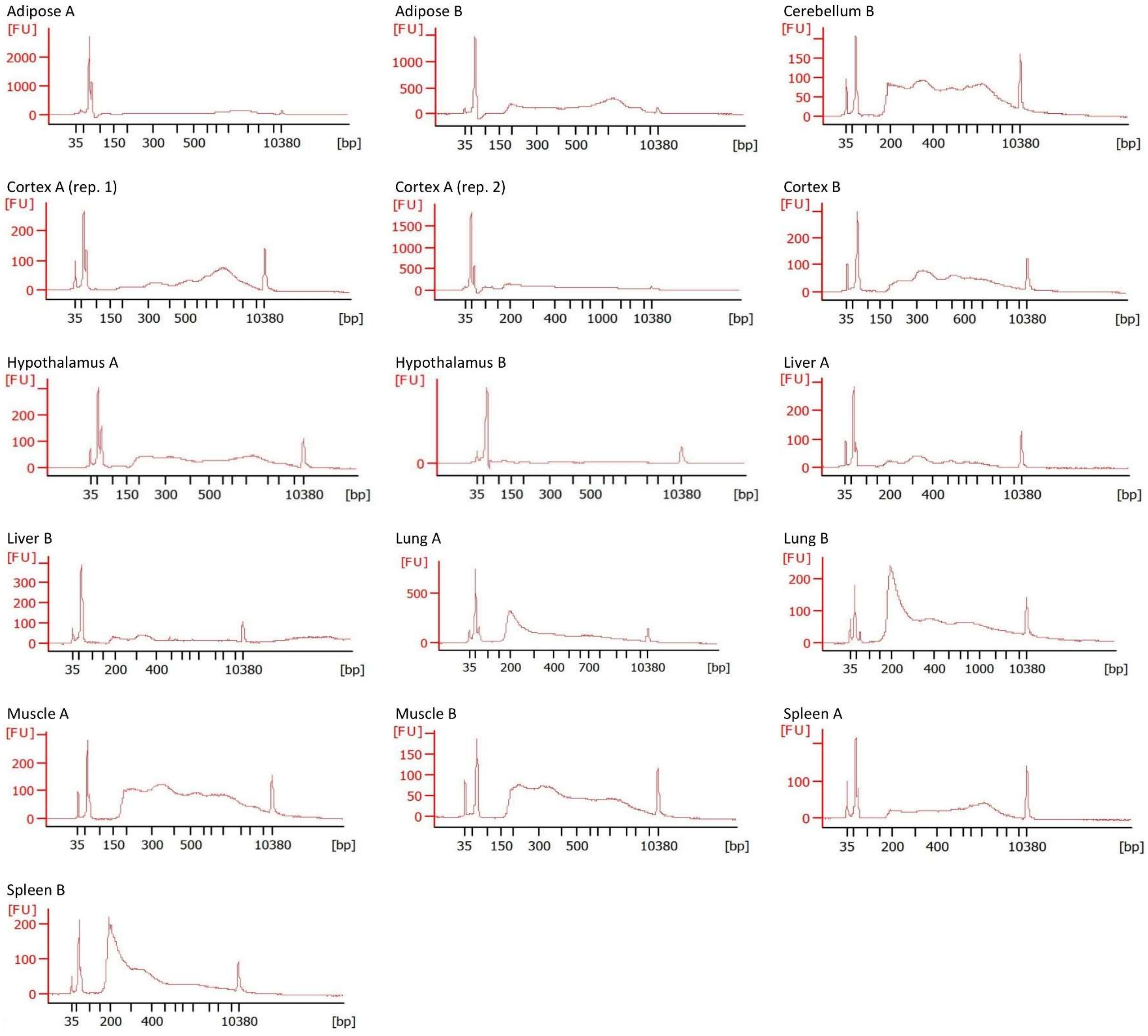

Figure S9. Bioanalyzer traces of cattle ATAC-seq libraries prior to size selection. Bioanalyzer traces were used to check for nucleosomal laddering. Size selection removed excess primer and fragments > 250 bp.

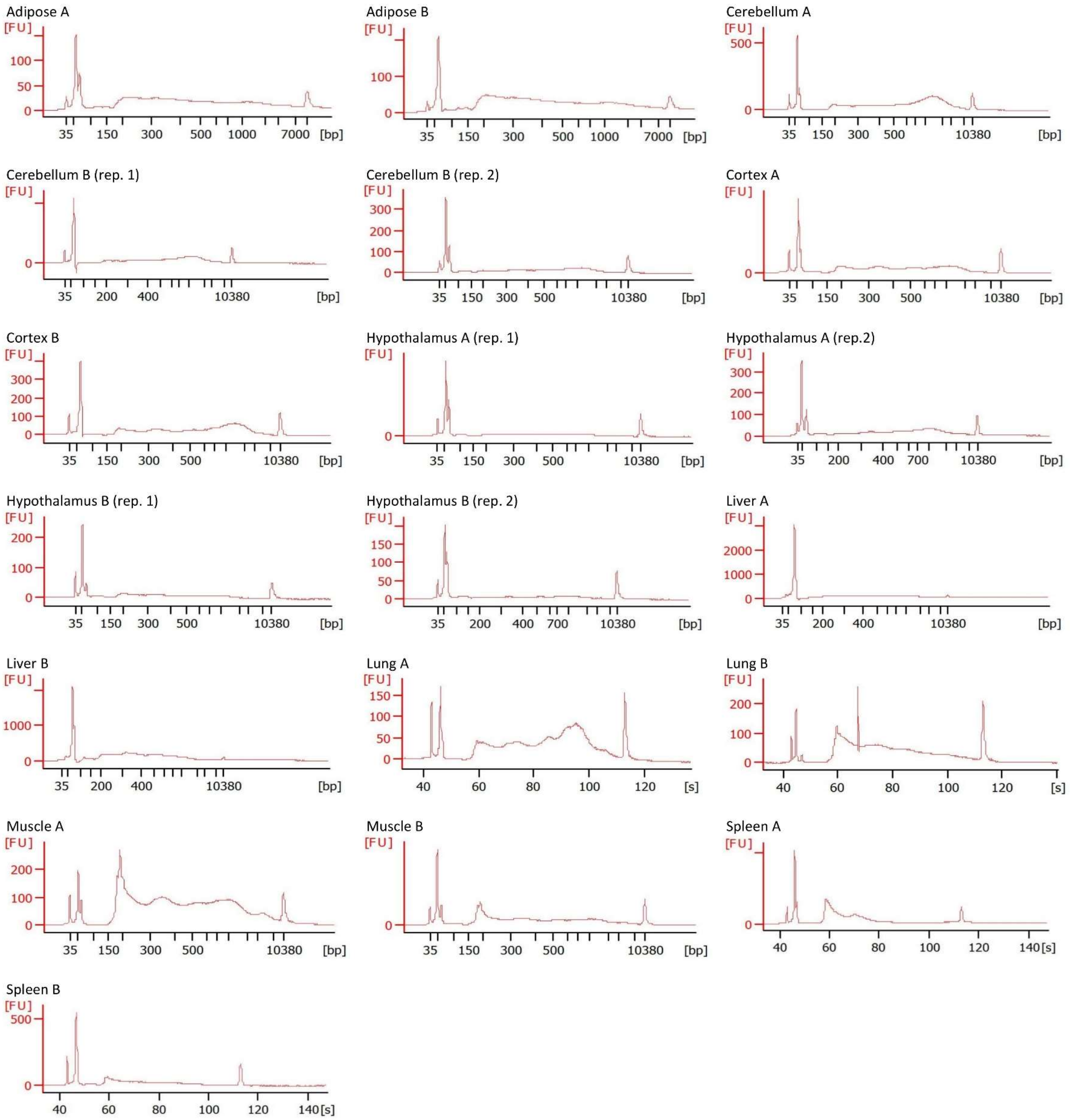

Figure S10. Bioanalyzer traces of pig ATAC-seq libraries prior to size selection. Bioanalyzer traces were used to check for nucleosomal laddering. Size selection removed excess primer and fragments > 250 bp.

Table S1. ATAC-seq library construction details. For each library constructed, rounds of PCR amplification, number of cells used as input, and concentration in the 150-250 bp range prior to size selection are indicated.

Species	Tissue	Biological replicate	Technical replicate	PCR cycles	Cell input	150-250 bp range Concentration (ng/ul)	150-250 bp range Total DNA (ng)
Cattle	Adipose	A	1	10	50,000	0.51	5.08
Cattle	Adipose	B	1	10	200,000	1.57	15.69
Cattle	Cerebellum	B	1	12	500,000	3.27	32.71
Cattle	Cerebellum	B	1	12	500,000	3.27	32.71
Cattle	Cortex	A	1	12	200,000	0.55	5.48
Cattle	Cortex	A	2	13	1,000,000	2.00	19.99
Cattle	Cortex	B	1	12	200,000	1.57	15.71
Cattle	Hypothalamus	A	1	11	500,000	1.95	19.52
Cattle	Hypothalamus	B	1	11	500,000	0.22	2.17
Cattle	Liver	A	1	12	50,000	1.46	14.62
Cattle	Liver	B	1	12	500,000	1.77	17.68
Cattle	Lung	A	1	12	50,000	12.98	129.78
Cattle	Lung	B	1	12	50,000	7.37	73.67
Cattle	Muscle	A	1	12	100,000	4.15	41.53
Cattle	Muscle	B	1	12	70,000	4.08	40.84
Cattle	Spleen	A	1	12	50,000	1.17	11.67
Cattle	Spleen	B	1	12	50,000	9.59	95.86
Pig	Adipose	A	1	12	500,000	3.42	34.22
Pig	Adipose	B	1	12	500,000	4.52	45.24
Pig	Cerebellum	A	1	10	200,000	1.28	12.85
Pig	Cerebellum	B	1	10	500,000	0.91	9.09
Pig	Cerebellum	B	2	11	200,000	0.77	7.66
Pig	Cortex	A	1	10	200,000	1.48	14.75
Pig	Cortex	B	1	10	200,000	1.53	15.34
Pig	Hypothalamus	A	1	12	500,000	0.47	4.68
Pig	Hypothalamus	A	2	11	500,000	0.90	8.98
Pig	Hypothalamus	B	1	12	500,000	1.41	14.10
Pig	Hypothalamus	B	2	11	500,000	0.37	3.65
Pig	Liver	A	1	10	200,000	0.71	7.06
Pig	Liver	B	1	10	500,000	2.11	21.08
Pig	Lung	A	1	10	200,000	2.00	20.00
Pig	Lung	B	1	10	500,000	3.04	30.36
Pig	Muscle	A	1	10	500,000	6.26	62.62
Pig	Muscle	B	1	10	500,000	3.16	31.65
Pig	Spleen	A	1	10	500,000	7.04	70.41
Pig	Spleen	B	1	10	200,000	2.34	23.40

Table S2. Functional annotation clustering of genes with conserved and global TSS accessibility. Genes with accessible TSS (± 50 bp) in all profiled tissues in all species were subjected to functional annotation clustering to identify enriched cellular functions. Top four clusters reported.

Annotation Cluster 1	*Enrichment Score: 6.83*	*Genes*	*p-value*	*Benjamini*
GOTERM_BP_5	DNA repair	54	6.50E-09	5.50E-06
GOTERM_BP_5	DNA metabolic process	85	1.60E-07	5.90E-05
GOTERM_BP_5	DNA recombination	31	3.20E-06	7.90E-04
Annotation Cluster 2	*Enrichment Score: 5.44*	*Genes*	*p-value*	*Benjamini*
GOTERM_BP_5	cellular protein metabolic process	322	4.80E-09	5.50E-06
GOTERM_BP_5	cellular protein modification process	245	5.00E-07	1.40E-04
GOTERM_BP_5	protein modification process	245	5.00E-07	1.40E-04
GOTERM_BP_5	regulation of protein modification process	96	1.40E-01	8.50E-01
Annotation Cluster 3	*Enrichment Score: 4.18*	*Genes*	*p-value*	*Benjamini*
GOTERM_CC_DIRECT	chromosome	42	4.10E-07	3.20E-05
GOTERM_CC_DIRECT	chromosome, centromeric region	21	3.30E-05	1.50E-03
GOTERM_CC_DIRECT	kinetochore	17	4.00E-04	1.50E-02
GOTERM_CC_DIRECT	condensed chromosome kinetochore	12	3.60E-03	8.60E-02
Annotation Cluster 4	*Enrichment Score: 3.82*	*Genes*	*p-value*	*Benjamini*
GOTERM_BP_5	mitotic sister chromatid segregation	22	8.30E-06	1.50E-03
GOTERM_BP_5	sister chromatid segregation	24	1.60E-05	2.60E-03
GOTERM_BP_5	mitotic metaphase plate congression	9	3.20E-04	2.90E-02
GOTERM_BP_5	establishment of chromosome localization	12	7.30E-04	5.40E-02
GOTERM_BP_5	metaphase plate congression	9	2.60E-03	1.30E-01

Table S3. Functional annotation clustering of genes near conserved intergenic open chromatin. Genes that were closest (within 100kb) to intergenic open chromatin that was conserved in all three species were subjected to functional annotation clustering to identify enriched cellular functions. Top four clusters reported.

Annotation Cluster 1	*Enrichment Score: 5.93*	*Genes*	*p-value*	*Benjamini*
GOTERM_BP_5	nervous system development	41	1.30E-07	2.60E-04
GOTERM_BP_5	central nervous system development	24	9.50E-07	9.30E-04
GOTERM_BP_5	brain development	19	1.30E-05	4.30E-03
Annotation Cluster 2	*Enrichment Score: 3.48*	*Genes*	*p-value*	*Benjamini*
GOTERM_BP_5	mesenchymal cell development	9	5.20E-05	1.10E-02
GOTERM_BP_5	mesenchymal cell differentiation	9	8.70E-05	1.20E-02
GOTERM_BP_5	mesenchyme development	10	1.10E-04	1.40E-02
GOTERM_BP_5	ameboidal-type cell migration	11	3.00E-04	2.50E-02
GOTERM_BP_5	neural crest cell migration	5	9.10E-04	4.80E-02
GOTERM_BP_5	central nervous system neuron differentiation	8	1.10E-03	5.40E-02
GOTERM_BP_5	neural crest cell development	5	2.90E-03	1.10E-01
Annotation Cluster 3	*Enrichment Score: 3.26*	*Genes*	*p-value*	*Benjamini*
GOTERM_BP_5	regionalization	12	7.70E-05	1.10E-02
GOTERM_BP_5	embryo development ending in birth or egg hatching	17	1.50E-04	1.50E-02
GOTERM_BP_5	neural tube development	6	1.50E-02	2.60E-01
Annotation Cluster 4	*Enrichment Score: 2.78*	*Genes*	*p-value*	*Benjamini*
GOTERM_BP_5	organ morphogenesis	24	3.10E-06	1.50E-03
GOTERM_BP_5	cell migration	22	2.60E-04	2.30E-02
GOTERM_BP_5	ameboidal-type cell migration	11	3.00E-04	2.50E-02
GOTERM_BP_5	cell development	33	3.30E-04	2.50E-02
GOTERM_BP_5	circulatory system development	20	4.10E-04	2.70E-02
GOTERM_BP_5	cardiovascular system development	20	4.10E-04	2.70E-02
GOTERM_BP_5	heart development	14	6.70E-04	3.80E-02
GOTERM_BP_5	vasculature development	13	5.40E-03	1.60E-01
GOTERM_BP_5	blood vessel development	12	9.50E-03	2.10E-01
GOTERM_BP_5	positive regulation of cell migration	9	2.00E-02	3.10E-01
GOTERM_BP_5	positive regulation of cell motility	9	2.40E-02	3.20E-01
GOTERM_BP_5	positive regulation of cellular component movement	9	2.70E-02	3.40E-01
GOTERM_BP_5	angiogenesis	7	1.20E-01	6.50E-01

Table S4. ATAC-seq oligos used for PCR. Sequences have been previously described by Buenrostro *et al*, 2013. Primers 2A-2X contain variable barcodes which permit library pooling prior to sequencing, and which were used to demultiplex sequencing data.

Primer	*Sequence (5' to 3')*
1	AATGATACGGCGACCACCGAGATCTACACTCGTCGGCAGCGTCAGATGTG
2A	CAAGCAGAAGACGGCATACGAGATCTAGTACGGTCTCGTGGGCTCGGAGATGT
2B	CAAGCAGAAGACGGCATACGAGATTTCTGCCTGTCTCGTGGGCTCGGAGATGT
2C	CAAGCAGAAGACGGCATACGAGATGCTCAGGAGTCTCGTGGGCTCGGAGATGT
2D	CAAGCAGAAGACGGCATACGAGATAGGAGTCCGTCTCGTGGGCTCGGAGATGT
2E	CAAGCAGAAGACGGCATACGAGATCATGCCTAGTCTCGTGGGCTCGGAGATGT
2F	CAAGCAGAAGACGGCATACGAGATGTAGAGAGGTCTCGTGGGCTCGGAGATGT
2G	CAAGCAGAAGACGGCATACGAGATCCTCTCTGGTCTCGTGGGCTCGGAGATGT
2H	CAAGCAGAAGACGGCATACGAGATAGCGTAGCGTCTCGTGGGCTCGGAGATGT
2I	CAAGCAGAAGACGGCATACGAGATCAGCCTCGGTCTCGTGGGCTCGGAGATGT
2J	CAAGCAGAAGACGGCATACGAGATTGCCTCTTGTCTCGTGGGCTCGGAGATGT
2K	CAAGCAGAAGACGGCATACGAGATTCCTCTACGTCTCGTGGGCTCGGAGATGT
2L	CAAGCAGAAGACGGCATACGAGATATCACGACGTCTCGTGGGCTCGGAGATGT
2M	CAAGCAGAAGACGGCATACGAGATACAGTGGTGTCTCGTGGGCTCGGAGATGT
2N	CAAGCAGAAGACGGCATACGAGATCAGATCCAGTCTCGTGGGCTCGGAGATGT
2O	CAAGCAGAAGACGGCATACGAGATACAAACGGGTCTCGTGGGCTCGGAGATGT
2P	CAAGCAGAAGACGGCATACGAGATACCCAGCAGTCTCGTGGGCTCGGAGATGT
2Q	CAAGCAGAAGACGGCATACGAGATAACCCCTCGTCTCGTGGGCTCGGAGATGT
2R	CAAGCAGAAGACGGCATACGAGATCCCAACCTGTCTCGTGGGCTCGGAGATGT
2S	CAAGCAGAAGACGGCATACGAGATCACCACACGTCTCGTGGGCTCGGAGATGT
2T	CAAGCAGAAGACGGCATACGAGATGAAACCCAGTCTCGTGGGCTCGGAGATGT
2U	CAAGCAGAAGACGGCATACGAGATTGTGACCAGTCTCGTGGGCTCGGAGATGT
2V	CAAGCAGAAGACGGCATACGAGATAGGGTCAAGTCTCGTGGGCTCGGAGATGT
2W	CAAGCAGAAGACGGCATACGAGATAGGAGTGGGTCTCGTGGGCTCGGAGATGT
2X	CAAGCAGAAGACGGCATACGAGATTCGCCTTAGTCTCGTGGGCTCGGAGATGT
